# Comparing the response of the indigenous microbial community to crude oil amendment in oxic versus hypoxic conditions

**DOI:** 10.3389/frmbi.2023.1270352

**Published:** 2023-12-14

**Authors:** Z. G. Griffiths, Andrew D. Putt, J. I. Miller, Maria Fernanda Campa, Dominique C. Joyner, O. Pelz, Nargiz Garajayeva, M. Ceccopieri, P. Gardinali, Terry C. Hazen

**Affiliations:** ^1^ Bredesen Center – Genome Science & Technology, University of Tennessee, Knoxville, TN, United States; ^2^ Institute for a Secure and Sustainable Environment, University of Tennessee, Knoxville, TN, United States; ^3^ Oak Ridge National Laboratory, Oak Ridge, TN, United States; ^4^ Department of Earth & Planetary Sciences, University of Tennessee, Knoxville, TN, United States; ^5^ Office of Innovative Technologies, University of Tennessee, Knoxville, TN, United States; ^6^ Department of Civil and Environmental Engineering, University of Tennessee, Knoxville, TN, United States; ^7^ Regulatory Compliance and Environment, BP AGT Region, Baku, Azerbaijan; ^8^ Environmental Analysis Research Lab, Florida International University, Miami, FL, United States

**Keywords:** oil biodegradation, temperature, oxic, hypoxic, marine, hydrocarbons

## Abstract

**Introduction:**

The Caspian Sea is the world’s largest landlocked saline lake which lies between Europe and Asia. This region is particularly known for its large-scale oil reserves, pipelines, and drilling activities, which have contributed to the environmental decline of this lake. In addition to pollution from the petroleum industry, drainage from various river basins brings an influx of residential, industrial, and agricultural effluents that induce eutrophication and hypoxic conditions in deeper, colder waters, creating an oxygen gradient. The temperature and oxygen stratification in this environment has presented a unique opportunity to investigate the potential of the biodegradative processes carried out by the indigenous microbial community. We believe these indigenous microbes possess different metabolic capabilities to degrade oil as they adapted to declining oxygen concentrations and temperatures with increasing depths over a prolonged period. Hence, community structure and composition will vary with depth.

**Methods:**

Microcosms were set up to observe the indigenous microbial reaction after a 60 ppm native crude oil amendment over 115 days. Surface water microcosms were incubated at 28ºC and aerated while deep water microcosms were incubated at 8ºC under anaerobic conditions. These two environmental conditions represent the temperature and oxygen extremes along the gradient and were selected as we try to simulate the indigenous community’s response to this oil contamination. DNA was extracted and amplified from these microcosms and sequenced. Bioinformatic analysis was performed to track changes in the abundance of taxa present and biodiversity over different time points to show the progression of community structure.

**Results:**

All microcosms showed the presence of hydrocarbon-degrading phyla, whose presence is consistent with other reports from oil-enriched environments. However, distinct communities were observed in oxic versus hypoxic microcosms.

**Conclusion:**

Orders of *Bacteria* related to sulfate and nitrogen cycling were found in hypoxic microcosms, indicating a possible mechanism for the anaerobic biodegradation of crude oil. GC-MS analysis of initial and final microcosms also provided evidence of degradation of hydrocarbon fractions in both warm, oxic and cold, hypoxic conditions.

## 1 Introduction

**Figure 1:**
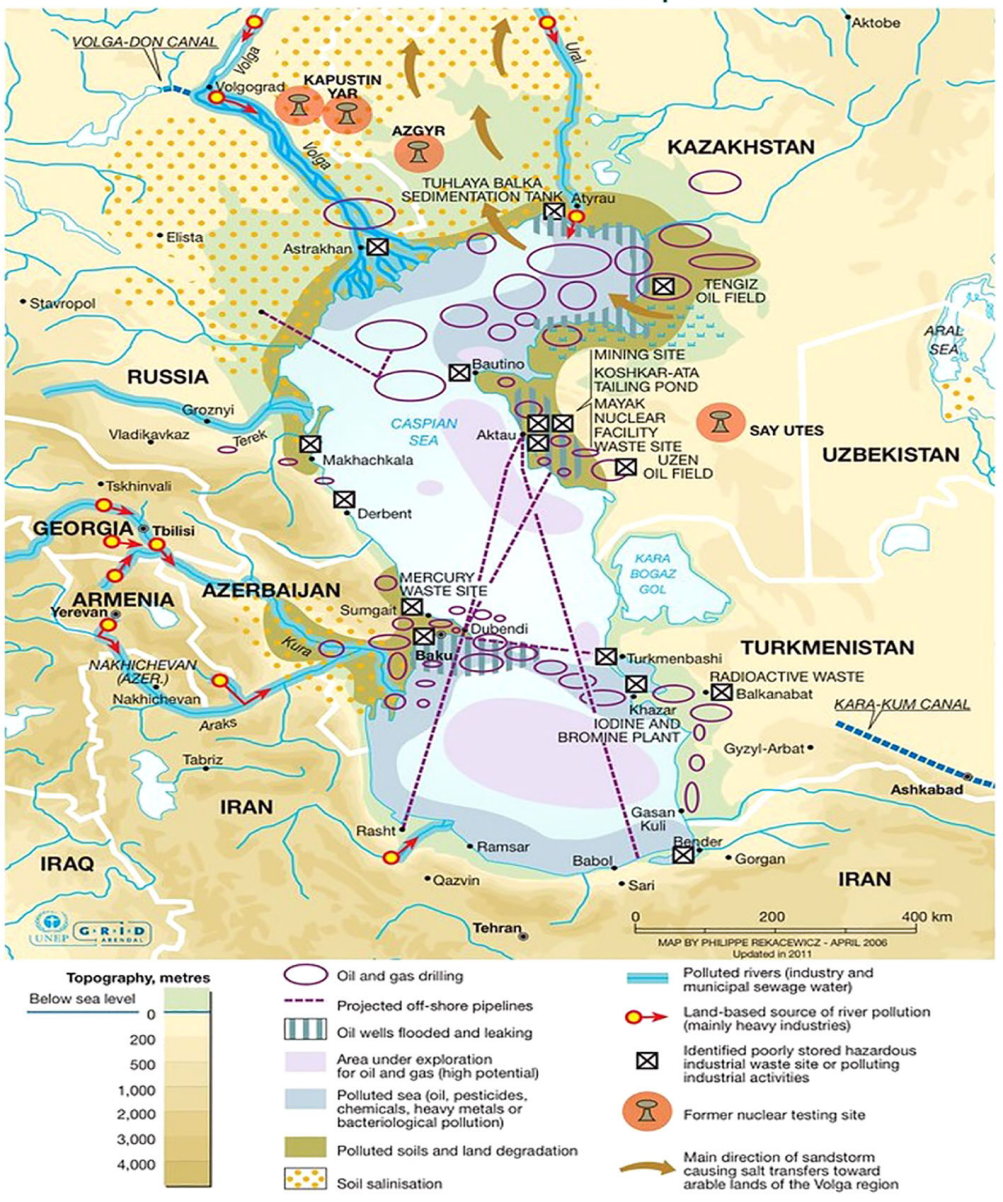
Showing environmental hazards in the Caspian Sea. Industrial activities, especially oil extraction, are the main sources of water and land pollution in the region. Off-shore oil extraction, pipeline transport and oil refineries have generated large quantities of toxic waste and oil contamination that remains trapped within the Caspian Sea because of its endorheic nature. Philippe Rekacewicz (le Monde Diplomatique) assisted by Laura Margueritte and Cecile Marin (2012).

The Caspian Sea is the world’s largest brackish lake with a salinity of approximately one-third of normal seawater (Leroy et al., 2007; Owen et al., 2023). This lake lies between Europe and Asia and borders five (5) countries along its coast – Russia, Azerbaijan, Iran, Kazakhstan, and Turkmenistan (**Figure 1**). The Caspian Sea is an endorheic lake with no outlets or direct connection to an ocean; however, more than 130 rivers flow into the Caspian Sea (Owen et al., 2023). The Caspian Sea is divided into three main geological basins – the northern, middle, and southern basins. Water depths increase towards the southern end of this basin, reaching a maximum depth of 1025 meters (Dumont et al., 2004; EIA, 2013; Owen et al., 2023).

The Caspian basin is one of the oldest oil-producing regions in the world (Effimoff, 2000; Katz et al., 2000). This region is well known for its considerable oil reserves with an estimated 48 billion barrels of oil and 292 trillion cubic feet of natural gas in proven and probable reserves (EIA, 2013; Holstein et al., 2018). The Caspian basin also contributes to the global energy supply with an average production of 2.6 million barrels/day according to the US Energy Information Administration EIA (2013). Azeri light crude oil is extracted offshore near Azerbaijan’s shoreline of the Caspian Sea (**Figure 1**). It is a well-balanced, sweet (low sulfur content) crude that is valued since a greater percentage of it can be easily refined into desirable petroleum products, for example naphtha (for solvents), gasoline and other fuel oils. The large scale of this industry has contributed to the waterbody’s environmental decline. This region faces challenges with the transportation of the oil extracted and produced since its hydrocarbon reserves are relatively far from export markets. These long distances require shipping and pipeline infrastructure to transport oil which a significant source of oil pollution (Holstein et al., 2018). Crude oil is both a natural and anthropogenic contaminant within the Caspian Sea. Oil also enters the Caspian through natural seepages from the seabed and mud volcanoes (Mityagina et al., 2019). Approximately one million tons of this oil leaked into the Caspian annually from these various sources (Mityagina et al., 2019; Modabberi et al., 2020). The Caspian Sea is surrounded by urban, industrial, and agricultural areas. In addition to oil pollution from the petroleum industry, drainage from various river basins brings an influx of untreated sewage, industrial and agricultural effluents that induce eutrophication which causes anoxic (low oxygen) conditions in deeper, colder waters (Miller et al., 2020; Modabberi et al., 2020). Oxygen saturation has dropped to around ~3% in deeper waters (**Supplementary Table 1**). Such anoxic conditions affect the mortality of benthic marine organisms and alter biogeochemical cycling (Aladin and Plotnikov, 2004). Since the Caspian Sea is an enclosed waterbody, it acts as a reservoir for pollutants where they begin to accumulate, making it particularly vulnerable to human influence. The lack of outlets, weak tidal forces, and increasing evaporation rates encourage the accumulation of pollutants in the waters and benthic sediments of the Caspian Sea (Nasrollahzadeh, 2010; Medvedev et al., 2017; Mishra et al., 2017).

**Figure 2:**
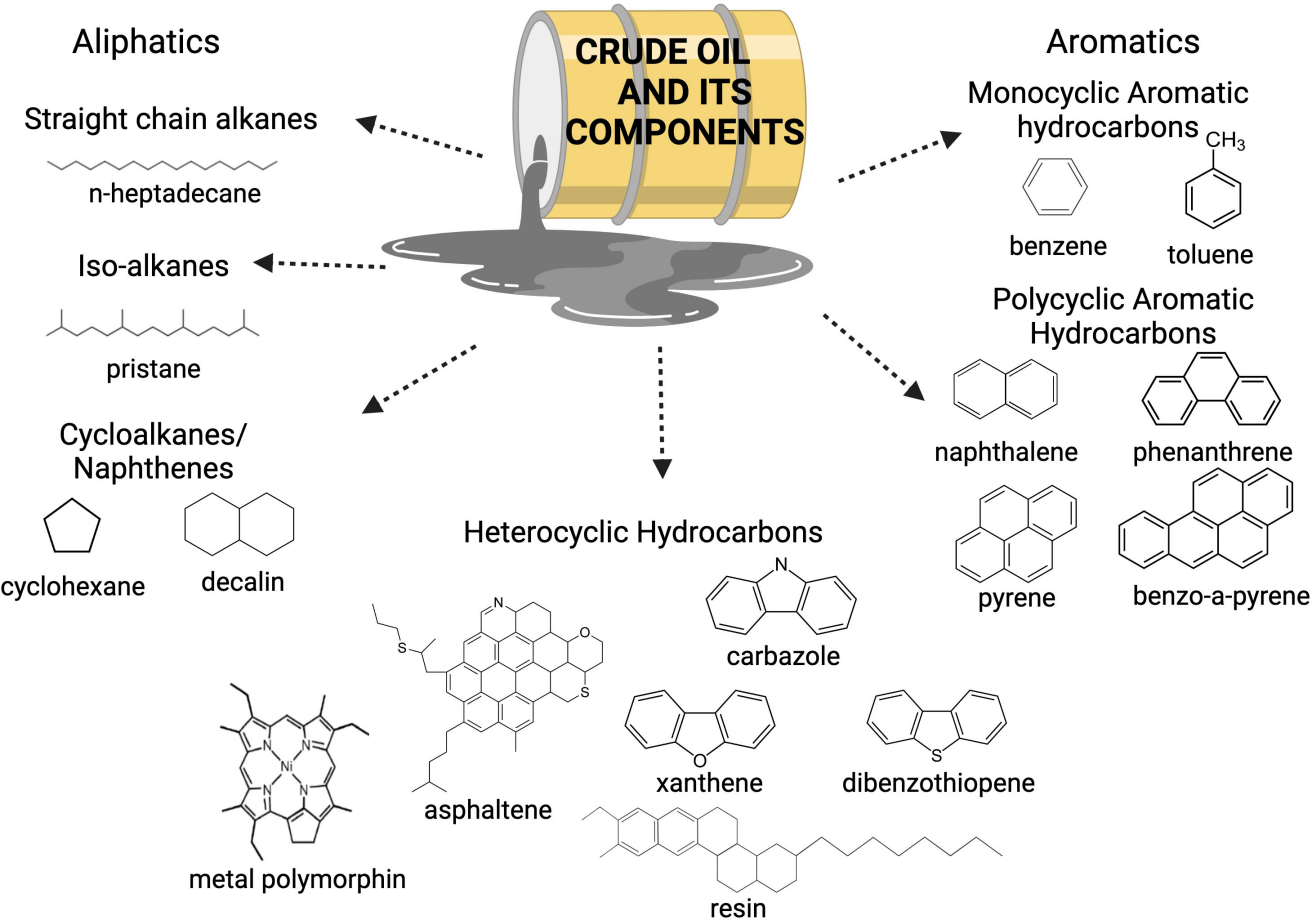
Crude oil is composed of different hydrocarbon fractions, a mix of metals and other compounds. As a result, crude oil has a complex chemistry. Adapted from Mahjoubi et al. (2018).

Crude oil is a complex mixture of thousands of organic compounds containing mainly hydrogen and carbon, which is highlighted in **Figure 2** (Hassanshahian and Cappello, 2013; Mahjoubi et al., 2018; Wang et al., 2021). Depending on their chemical structure, these hydrocarbons can be classified into alkanes, alkenes, cycloalkanes, and aromatics (Stauffer et al., 2008). Crude oil also contains derivatives of nitrogen-, sulfur- and oxygen-containing compounds (Morgan and Watkinson, 1994; Stauffer et al., 2008). Metals and other inorganic compounds may also be present at very low concentrations. The complex chemistry of crude oil needs a complex community of microorganisms to degrade it since each component has a distinct chemical property that influences its biodegradability. No single organism is able to metabolize all hydrocarbon fractions in crude oil. Microbial degradation of the crude oil fractions follows an ease of degradation from bioavailable to recalcitrant compounds, typically in the order of n-alkanes > branched alkanes > aromatics > cyclic alkanes > polycyclic aromatic hydrocarbons > resins and asphaltenes (Das and Chandran, 2011; Varjani, 2017; Varjani and Upasani, 2017). This results in a succession of microbial bloom and death as compounds are consumed (Hazen et al., 2010; Olajire and Essien, 2014; Mahjoubi et al., 2018). Hence, these microbes are working in conjunction with each other with their varying enzymatic capabilities to provide a dynamic community response to this contaminant.

**Figure 3:**
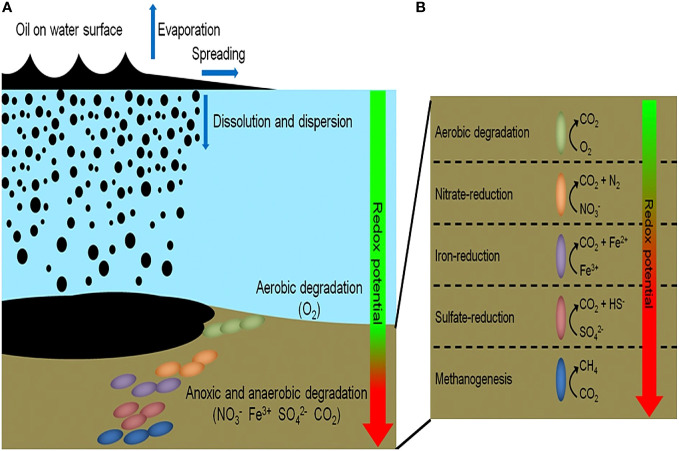
**(A, B)** Showing the fate of oil spills in a marine ecosystem. Aerobic biodegradation will proceed in surface waters but as oxygen concentration decreases with depth, so does the rate of aerobic biodegradation. Anaerobic biodegradation will proceed based on the redox potential of available compounds at deeper depths. Source: (Daghio et al., 2017).

**Figure 3** illustrates the changing biochemical demands of oil biodegradation with decreasing oxygen concentrations. Alternate electron acceptors (NO_3_^−^, Fe^3+^, SO_4_^2−^, CO_2_) are needed to complete this process in the absence of oxygen (Hazen et al., 2016). Other environmental variables, such as temperature, can also influence oil biodegradation. Temperature normally affects the metabolic rate of these hydrocarbon-degrading organisms with metabolism decreasing with decreasing temperatures (Das and Chandran, 2011). However, over long periods of time, changes in enzymatic ability to degrade at low temperatures will increase and the organisms will become psychrophiles and will degrade oil faster at colder temperatures (Hazen et al., 2010; Hazen, 2018; Brown et al., 2020). The physicochemical characteristics of the oil are also affected by temperature where oil viscosity increases with decreasing temperature (Ribicic et al., 2018b). Hence, the biodegradation will proceed differently in surface waters (warmer, well-oxygenated) versus deep waters (cold, anoxic conditions) of the Caspian Sea.

### 1.1 Aerobic vs. anaerobic biodegradation of crude oil

**Figure 4:**
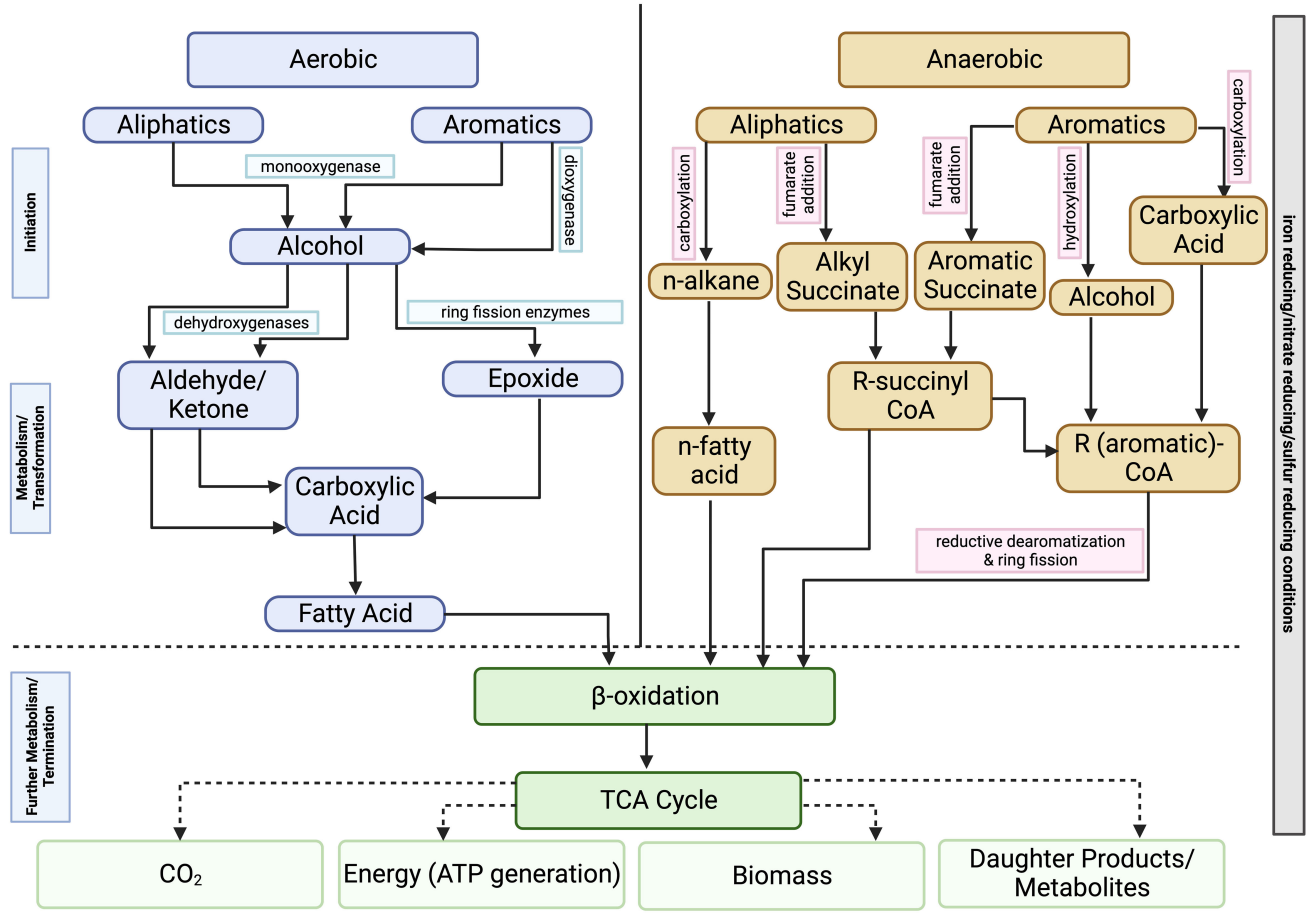
Pathways for the aerobic and anaerobic biodegradation of hydrocarbon compounds.

Bacteria can biodegrade the hydrocarbon fractions found in crude oil in both aerobic and anaerobic conditions (**Figure 4**). This process will usually proceed more quickly in aerobic conditions. As previously mentioned, the biochemical demands of hydrocarbon degradation will vary with oxygen concentration. In aerobic conditions, oxygen is needed, both as a terminal electron acceptor and an activator, to begin hydrocarbon degradation. Metabolism of aliphatic hydrocarbons is usually initiated by the action of different oxidase and peroxidase enzymes to form a primary alcohol (Stroud et al., 2007; Sierra-Garcia et al., 2017). Further oxidation occurs, converting the hydrocarbons into fatty acids and other intermediate products that can be further utilized in the tricarboxylic acid (TCA) cycle (Hassanshahian et al., 2012). Aromatic hydrocarbons undergo a similar process where oxygen is introduced into the ring structure via the action of oxygenase enzymes. The ring is then cleaved, and there is further transformation of the intermediate products which are eventually passed into the TCA cycle (Hassanshahian et al., 2012; Varjani, 2017; Perez-Pantoja et al., 2019).

Anaerobic respiration proceeds differently with a reductive reaction. It is often a much slower process as it is more difficult to overcome the initial energy barrier of breaking the C-H bond (Boll and Heider, 2010; Ghosal et al., 2016; Mahjoubi et al., 2018). Organisms will generate energy by either coupling hydrocarbon oxidation to respiration via the reduction of an alternate terminal electron acceptor (e.g., nitrate, sulfate, iron) or using a fermentation pathway (Wartell et al., 2021). When the concentration of terminal electron acceptors has been exhausted, some microbes will use methanogenesis (using CO_2_ as terminal electron acceptor) to degrade various hydrocarbons. The complex hydrocarbons found in crude oil will be oxidized to methanogenic substrates, which can be further converted into methane (Laczi et al., 2020; Sengupta and Pal, 2021; Wartell et al., 2021). Different mechanisms have been proposed for how the initial attack of hydrocarbons proceeds in these conditions, but there is still a fair amount of uncertainty. **Figure 4** highlights some of these proposed mechanisms for initialization include the addition of hydrocarbon fractions to fumarate, methylation of unincorporated fractions, or hydroxylation with water (Sierra-Garcia et al., 2017; Wartell et al., 2021).

### 1.2 Tackling pollution with bioremediation

As a major oil production site, the Caspian region has been exposed to large pollution loads originating from oil and gas industries and natural seepages for a prolonged period (Nasrollahzadeh, 2010; Holstein et al., 2018; Mityagina et al., 2019). As a result, the microbial community within this region has had time to adapt to the presence of this pollutant through repeated exposure over thousands of years via natural selection. With this “memory response,” this indigenous community should be able to metabolize this contaminant and transform into less complex hydrocarbons, carbon dioxide, or microbial biomass via biodegradation.

Bioremediation presents a less invasive, environmentally friendly, and cost-effective option to tackle this oil pollution (Mahjoubi et al., 2018). As a result, it has been proposed as a strategy to combat rising oil pollution in the Caspian Sea over chemical or mechanical removal techniques (burning, skimming, dispersants), especially at increased depths. During oil spills, an influx of hydrocarbons enters the surface environment of the waterbody and into the oxygenated water column, where aerobic degradation can occur with oxygen as the final electron acceptor. However, oil escaping from natural seeps or leaking from underwater pipelines enters the environment at deeper depths into the low oxygen zone, where anaerobic degradation takes over, and alternate terminal electron acceptors are needed (**Figure 3**). This study aims to identify the unique community structure of the oxic versus the hypoxic environments and compare the biodegradative capabilities of each community in response to crude oil amendment. Understanding how the indigenous community responds in surface waters (warm, oxic conditions) versus deeper waters (cold, hypoxic, to anoxic conditions) will enable us to address difficulties associated with the biodegradation of crude oil.

## 2 Methods

### 2.1 Sample collection

Microcosm experiments were conducted using seawater collected from the Caspian Sea approximately 120 km east of Baku, Azerbaijan by British Petroleum (BP) Azerbaijan (**Figure 1**). Observation station 22 was designated as the sample collection site. The sediments at this site have consistently shown a high hydrocarbon content. This contamination is a result of a natural source, a mud volcano, and is unrelated to extraction activities (BP-Azerbaijan, 2019). Water was collected from ~25 m (near surface) and below the thermocline at ~350 m (near bottom) to represent oxic and hypoxic conditions, respectively. Seawater was collected in Niskin bottles affixed to a rosette carriage and brought onboard the ship for preservation. Samples were transferred to 1L amber bottles while minimizing exposure to the ambient environment and preserved at 4°C. Methods for sample collection were adapted from Miller et al. (2019).

### 2.2 Microcosm experiments for 16S rRNA analysis

Experiment microcosms were set up in triplicate and with 110 mL of seawater and inoculated with 60 ppm of Azeri crude oil provided by BP. Oxic microcosms were assembled under a Biosafety cabinet and shaken at 24°C, while hypoxic microcosms were assembled and sealed in an anaerobic chamber and then shaken at 8°C in the dark. This was done to represent the ambient environmental conditions of the collection sites (**Supplementary Table 1**). A control microcosm (in triplicates) comprised of seawater with no added crude oil was used to establish baseline conditions, representing no oil pollution. These microcosms were destructively sampled at 0 days, 5 days, 10 days, 15 days, and 115 days. Microcosm seawater was filtered through a 142 mm nylon filter with a pore size of 0.2 μm filter, and DNA was extracted from the filter using the Qiagen DNeasy PowerSoil extraction kit according to the manufacturer’s instructions (Valencia, CA). We targeted the V3-V4 hypervariable region of the 16s rRNA gene using the 515F and 806R primers developed by Caporaso et al. (2012) to amplify the extracted DNA using polymerase chain reaction (PCR) with Phusion Master Mix (Thermo Fisher Scientific, Waltham, MA). Samples were barcoded with a unique 12 base pair index to multiplex samples for sequencing. Library preparation for sequencing was done according to the standard Illumina-MiSeq protocol with a 300 cycle v2 reagent kit (San Diego, CA). Microcosms were set up in triplicates for statistical analysis and determination of significant differences between communities. FASTQ files for each replicate was uploaded to the NCBI short read archive under accession number PRJNA1026539.

### 2.3 Data analysis

Mothur v.1.46.1 (Schloss et al., 2009) was used for bioinformatic processing where sequences. Paired-end sequences were joined, trimmed, screened and aligned following the Mothur MiSeq standard operating procedure (https://mothur.org/wiki/miseq_sop/). Sequences were assigned to amplicon sequencing variants (ASVs) with Silva 138 as the reference database for taxonomic identification. Different statistical analyses were calculated using different data packages with R software. PERMANOVA analysis using the vegan package in R was performed to analyze differences in microbial community composition based on oxygen/temperature and oil amendment (Oksanen et al., 2022). A Wilcoxon test was performed to compare significant differences in genera in oxic versus hypoxic samples. An “indicator species analysis” was also done to identify any microbial species that were positively correlated to experimental conditions (De Cáceres et al., 2010).

### 2.4 Hydrocarbon analysis

Three experimental replicates (seawater amended with Azeri crude) for the initial (day 0) and final (day 115) time points were sent to the Environmental Analysis Research Lab at Florida International University. Samples (100 mL) were processed by liquid-liquid extraction in methylene chloride, concentrated to a final volume of 1 mL and analyzed via gas chromatography-mass spectrophotometry (GC-MS). Before the extraction, known amounts of the internal standard p-terphenyl-d_14_ and a mixture containing napthalene-d_8_, acenaphthene-d_10_, phenanthrene-d_10_, chrysene-d_12,_ and perylene-d_12_ were added. The *n*-alkanes from n-C_9_ to n-C_39_ and the isoprenoids pristane and phytane were determined by full scan acquisition. A selected ion monitoring method (SIM) was used for the analyses of the polycyclic aromatic hydrocarbons (PAHs), their alkylated homologs, and the C_30_-hopane triterpene. The retention times were determined using standard solutions containing all compounds of interest and quantification was based on the internal standards addition and calibration curves. All results are based on ratios to the recalcitrant biomarker C30-Hopane that should not be affected by significant degradation i.e., the normalized relative abundance.

## 3 Results

### 3.1 Community structure

**Figure 5:**
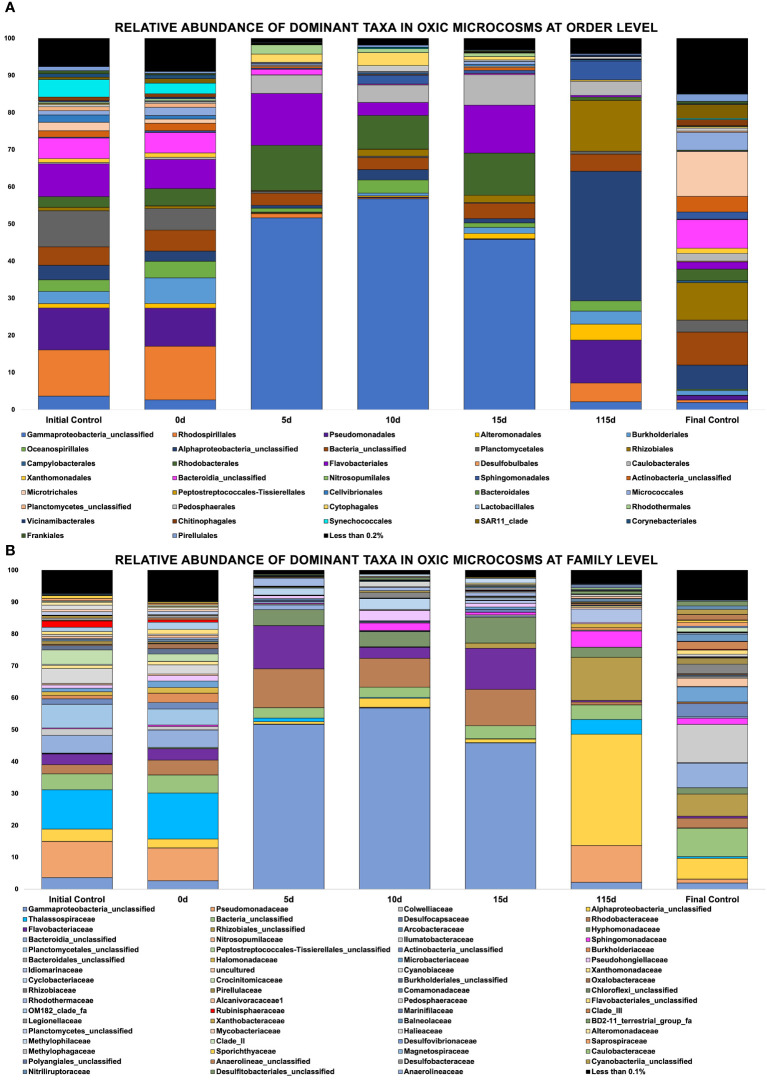
**(A, B)** Showing relative abundance of bacterial orders in oxic microcosms, **(B)** Showing relative abundance of bacterial families in oxic microcosms.

Community Analysis Two hundred and ninety (290) different phyla were identified across these oxic and hypoxic microcosms, with 29 phyla representing most of the biodiversity found in these samples. Oxic microcosms contained more phyla and displayed a greater biodiversity than their hypoxic counterparts, as indicated by the Shannon Diversity Index, which is an alpha-diversity metric. A greater biodiversity of microorganisms was observed in control microcosms than in oil-spiked microcosms. Oxic (**Figure 4**) and hypoxic (**Figure 5**) microcosms shared some similarities in the taxa present on Day 0 of the experiment and in their respective initial controls, but this distribution consistently changed as the experiment progressed.

**Figure 6:**
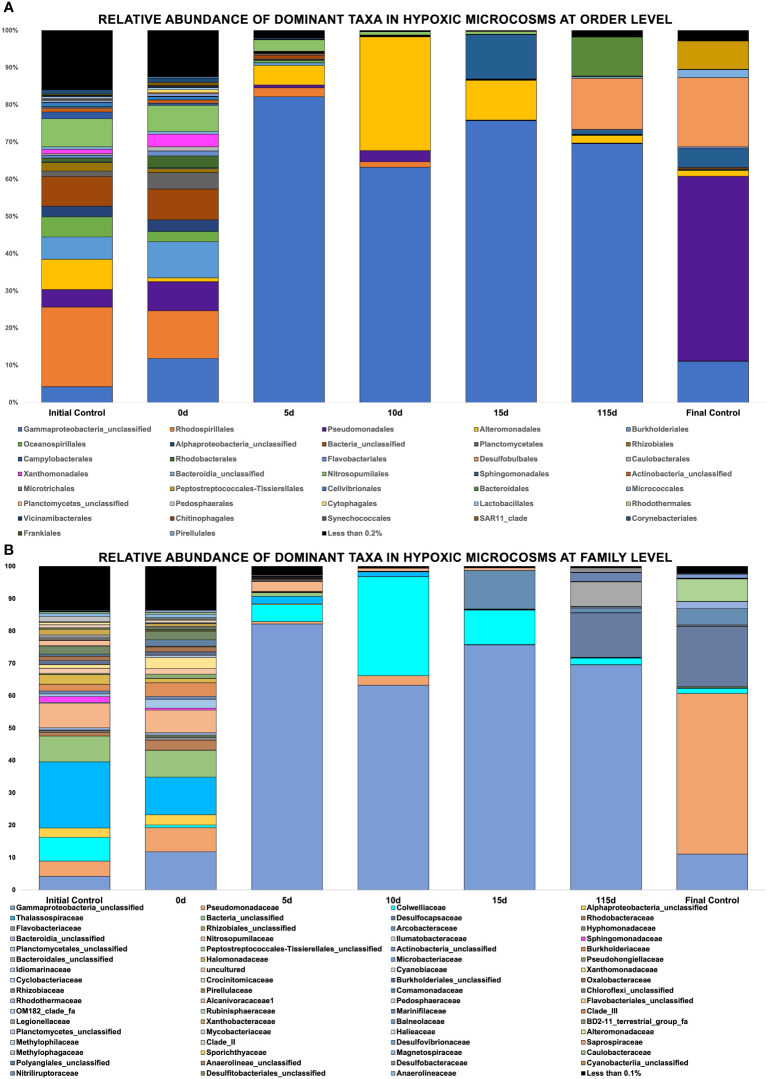
**(A, B)** Showing relative abundance of bacterial orders in hypoxic microcosms, Showing relative abundance of bacterial families in hypoxic microcosms.

The crude oil amendment caused a shift in the microbial community and significant differences were observed between oxic and hypoxic microcosms (p < 0.05). Additionally, the communities represented in the initial (day 0) and final (day 115) controls are vastly different from the communities observed in the experiment microcosms that were spiked with crude oil under oxic and hypoxic conditions (p ≤ 0.05). Different phyla were enriched by the presence of this contaminant, while others showed a decrease in their relative abundance when compared to the controls. There was also an observable pattern of sequential bloom and succession of microorganisms at different time points in both oxic and hypoxic conditions. Different ASVs of an unclassified *Gammaproteobacteria* was highly influenced by the presence of crude oil, as its relative abundance increased by 71% in hypoxic microcosms and 50% in oxic microcosms within the first five days (**Figures 5**, **6**). This taxon remained dominant in hypoxic microcosms until the final timepoint; however, in oxic microcosms, this dominance was diminished after day 15 when community diversity increased.

In oxic microcosms (**Figure 5**), the orders of *Rhodobacterales* and *Flavobacteriales* showed a relative increase in their abundances from the initial timepoint (day 0) up to day 15. However, their presence was diminished by day 115. The taxa *Caulobacter* seemed to be enriched by the oil amendment as well. The relative abundance of this taxa increased by approximately 7.5% from day 5 to day 15 of the experiment, and it was still present at the final timepoint (day 115). The presence of *Rhizobiales* and *Burkholderiales* increased slowly from day 10, with their highest abundances being observed at day 115. *Oceanospirallales* and *Pseudomondales* were temporarily impacted by the initial amendment of crude oil, and their abundance relatively decreased from day 0 to day 5. However, by day ten (10), their presence had rebounded and remained consistent until the final time point. The relative abundance of an unclassified *Alphaproteobacteria* remained relatively low for the experiment duration until the final time point, where it represented ~35% of the observed diversity. *Plantomycetales, Synechococcales, and Micrococcales* experienced a decline in their abundance over the experiment duration.

**Figure 6** shows the relative abundance of taxa found in hypoxic microcosms. The taxa *Alteromondales* was mainly found in hypoxic microcosms, and its relative abundance increased by 30% by day 10 in our experimental microcosms compared to the initial timepoint and the initial control. It then started to decrease in its abundance after this time, and by day 115, its relative abundance was similar to that of day 0. The presence of *Campylobacter* increased at later time points, and a bloom was observed on day 15 of the experiment. *Desulfobulbales* and *Bacteroidales* experienced a bloom on day 115 when compared to their negligible initial relative abundances. The taxa *Rhodospiralles* and *Nitrosopumilales* experienced a relative decline in their abundance from the initial time point until their disappearance after day 10.

### 3.2 Statistical analysis

**Figure 7:**
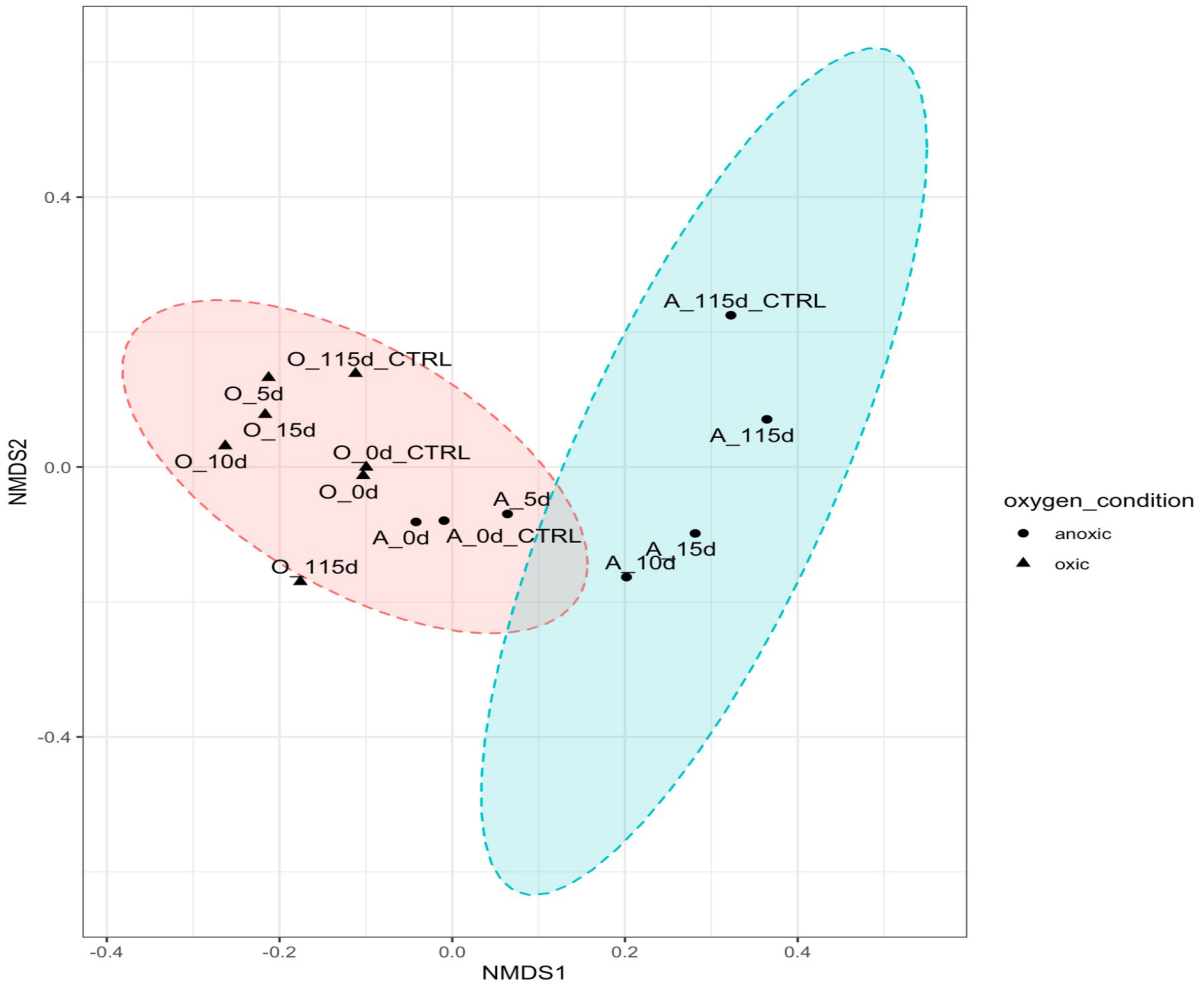
NMDS plotted with vegan package in R in 2 dimensions. Stress = 0.08444561. Oxic microcosms are denoted with a black triangle while hypoxic microcosms are represented by a black dot. Experiment microcosms are labelled with their corresponding day while control microcosms are labelled with their corresponding day_CTRL. Red and blue circles represent the 95% confidence intervals.

To further investigate differences in community structure, a pairwise Wilcoxon test (p < 0.05) was performed to evaluate significant differences in taxa present in oxic and hypoxic conditions. Microbes (*Sulfurimonas, MND1, Nitrosopumilaceae)* previously implicated in nitrogen and sulfur cycling seemed to be significantly correlated in hypoxic microcosms with the crude oil amendment. The presence of *Colwellia* and *Chloroflexi* were also more correlated with the low temperature, hypoxic microcosms. The difference in community structure between oxic and hypoxic microcosms was also shown using a NMDS plot with the Bray-Curtis dissimilarity matrix (**Figure 7**). While there is some similarity in initial time points, after day 5, there is a clear distinction between the communities in oxic and hypoxic microcosms. This plot also shows the similarity in communities from the initial controls and the day 0 time points. However, later time points show less similarity highlighting the influence of crude oil amendment on these communities as time progressed.

### 3.3 Indicator species analysis

The R code indicspecies developed by De Cáceres et al. (2010) was used to identify indicator species in the oxic (**Table 1**) and hypoxic (**Table 2**) microcosms amended with crude oil. *Colwellia* was once again highlighted as an important species in hypoxic microcosms as well as other taxa related to hydrocarbon degradation under deep-sea conditions. Indicator species identified in oxic microcosms also showed possible relation to hydrocarbon degradation in previous studies.

**Table 1:** Showing top 6 results of indicator species analysis in oxic microcosms (p < 0.05).

Genus	*statistic	Suspected Functional Role	Source
***Pseudooceanicola***	1	Reclassification from Oceanicola. Species from this taxon had previously been isolated from marine environments as aerobes or facultative anaerobes.	(Lai et al., 2015; Peeb et al., 2022)
***Phycisphaeraceae unclassified***	0.999	Taxa has been previously correlated to nitrogen removal/cycling and degradation of petroleum by-products/polymers.	(Liu et al., 2020)
***Litoricola***	0.999	Species within the Oceanospirillales family (which are typically classed as professional hydrocarbon degraders and have been found in other oil-contaminated environments, most notably after the Deepwater Horizon oil spill).	(Hazen et al., 2010; Doyle et al., 2018)
***Hyphomonas***	0.999	Taxon has been previously detected in oil-amended microcosms and mesocosms, suggesting possible involvement in hydrocarbon degradation.	(Kappell et al., 2014; Ribicic et al., 2018a)
***Bacteriovoracaceae unclassified***	0.999	Salt tolerant, predatory taxon that possibly regulates the structure of bacterial communities through predation.	(Pineiro et al., 2007; Welsh et al., 2016)
***Polyangiales unclassified***	0.999	Microbial predators with the capability to degrade complex molecules with hydrolytic enzymes, proteases, cellulase, etc.	(Nierychlo et al., 2020; Dueholm et al., 2022)

*A stat value of 1 signifies that this species was found in all samples within these microcosms.

**Table 2:** Showing top 6 results of indicator species analysis in hypoxic microcosms (p <0.05).

Genus	*statistic	Suspected Functional Role	Source
***WCHB1-41_ge***	1*	Suspected to contribute to arginine and fatty acid synthesis to provide energy for growth. Other bacteria within the order *Kiritimatiellae* have been associated with sulfated polysaccharides metabolism under anaerobic conditions.	(Spring et al., 2016; van Vliet et al., 2019; Guo et al., 2021).
***Sulfurimonas***	0.999	Mixotroph associated with deep-sea hydrothermal vents. Wang et al. (2020) isolated strains capable of utilizing n-alkanes and naphthalene and phenanthrene as a sole carbon source high pressure and low temperature.	(Wang et al., 2020)
***Spongiibacteraceae unclassified***	0.999	This taxon has been linked to aromatic and n-alkane hydrocarbon degradation in seawater at low temperatures and high pressures. Members have this taxon have also been implicated in biofilm formation on biopolymers in seawater.	(Ribicic et al., 2018b; Wang et al., 2020; Jacquin et al., 2021)
***Nitrosopumilaceae unclassified***	0.999	ammonia-oxidizing archaea implicated in nitrogen cycling and carbon fixation that has shown adaptations to low oxygen environments.	(Pester et al., 2011; Zhong et al., 2020)
***Patescibacteria*** ***unclassified***	0.998	Ultra-small bacteria with possible adaptations to low oxygen, low nutrient conditions in water environments. Related taxa found in sediments from the Marianas Trench with possible capability to metabolize organic compounds via fermentative processes and genes associated with respiratory nitrate reduction.	(Zhong et al., 2020; León-Zayas et al., 2017)
***Colwellia***	0.998	This taxon is cold adapted and capable of degrading short chain alkanes and possibly aromatic hydrocarbons. It was identified as a late bloomer found deep within the plume of the Deepwater Horizon oil spill.	(Redmond and Valentine, 2012; Mason et al., 2014)

*A stat value of 1 signifies that this species was found in all samples within these microcosms.

### 3.4 Hydrocarbon analysis

**Figure 8:**
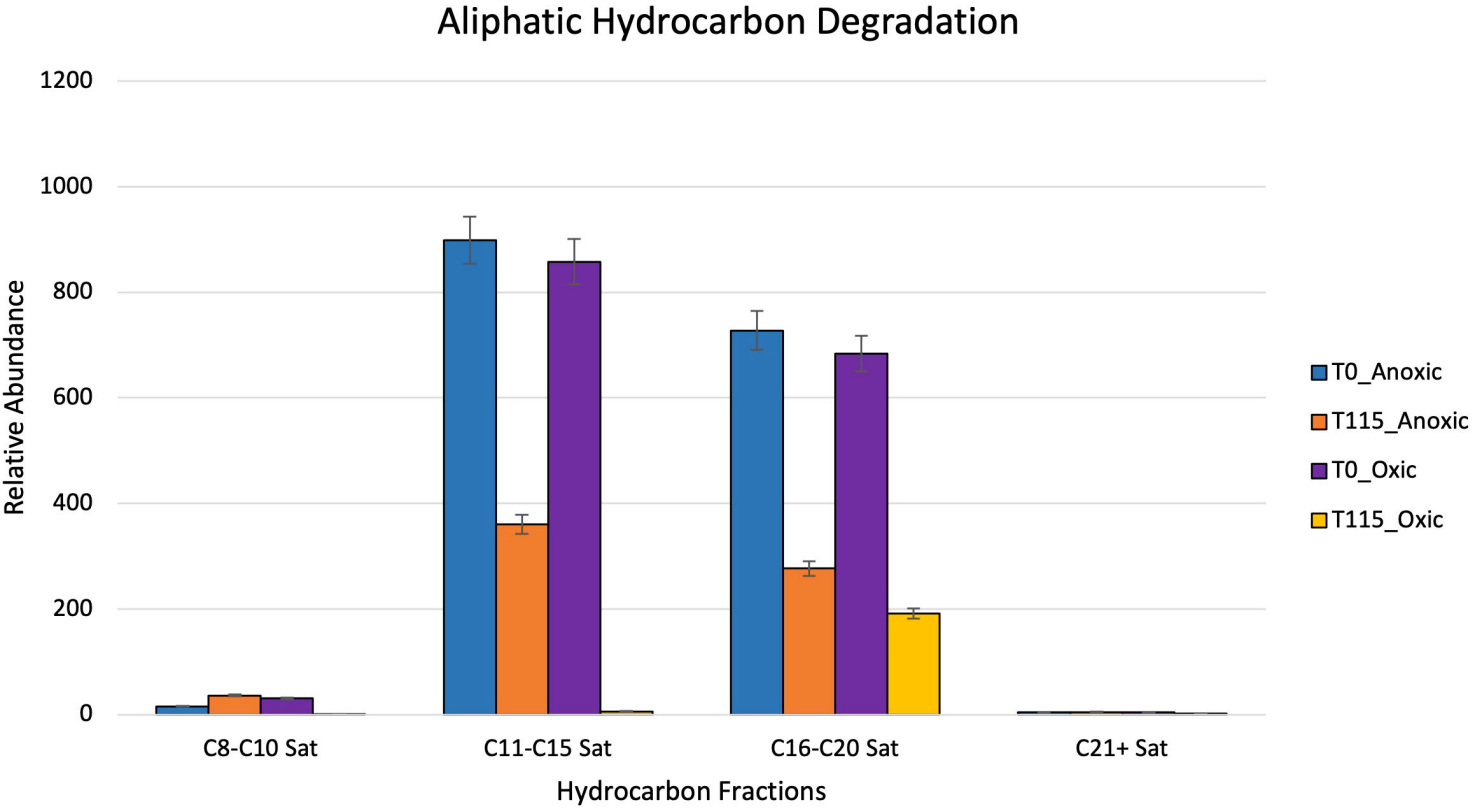
Showing relative abundance of aliphatic hydrocarbons measured by GC-MS in oxic and hypoxic microcosms. All results are semi quantitative and based on ratios to the recalcitrant biomarker C30-Hopane. Data is aggregated by groupings based on number of carbon atoms.

**Figure 9:**
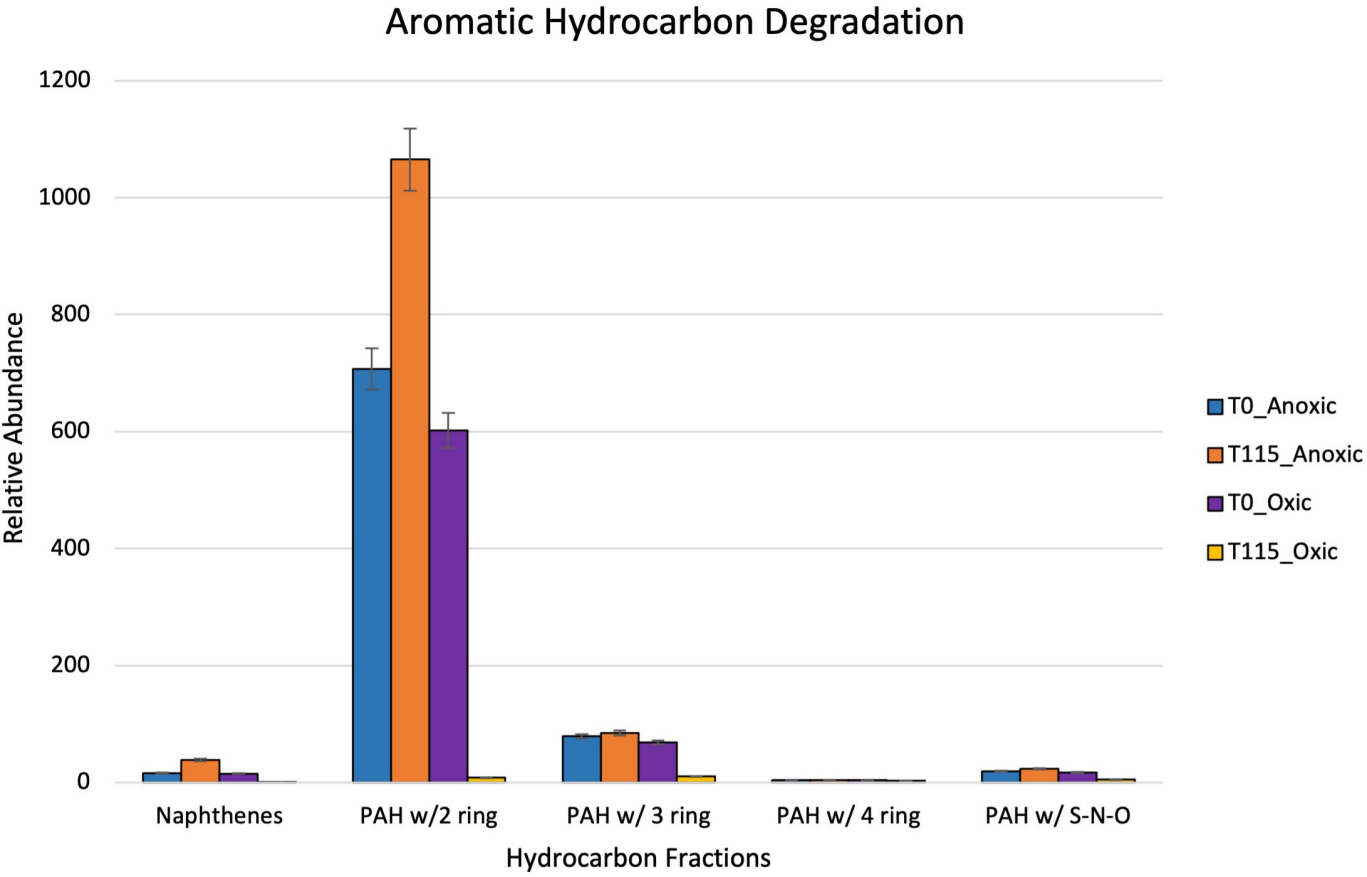
Showing relative abundance of aromatic hydrocarbons measured by GC-MS in oxic and hypoxic microcosms. All results are semi quantitative and based on ratios to the recalcitrant biomarker C30-Hopane. Data is aggregated by groupings based on number of rings.

After 115 days, oxic microcosms showed a change in chemical composition and a decrease in the relative concentration of the measurable hydrocarbons. Aliphatic hydrocarbons within the range C8-C14 were mainly depleted by day 115 (**Figure 8**). A similar trend was observed for aromatic hydrocarbons and cycloalkanes, with their disappearance being noted after 115 days (**Figure 9**). The hypoxic microcosms displayed a reduction in the relative abundance of aliphatic hydrocarbons with over 50% reduction in the total measured aliphatic hydrocarbons (**Figure 8**). The measured aromatic hydrocarbons and cycloalkanes showed a variation of responses. Naphthalene, its alkylated homologs, and the cycloalkane intermediates related to its degradation showed a noticeable increase in relative concentration after 115 days (8).

## 4 Discussion

This study aims to characterize the *in-situ* community structure of the Caspian Sea and to investigate the ability of the indigenous microbial community to degrade crude oil under oxic or hypoxic conditions. We observed changes in the community composition in response to crude oil amendment as well as a decrease in hydrocarbon fractions in both sets of microcosms. The environmental conditions (brackish water, oxygen/temperature gradient, eutrophication) in the Caspian Sea could potentially limit the extent of crude oil biodegradation (Varjani and Upasani, 2017). Hence, studying community structure and the potential “memory response” of these indigenous microbes will aid in elucidating their metabolic potential and adaptation to this pollutant.

Our seawater microcosms were kept at their ambient environmental conditions to potentially mimic the *in situ* temperature and oxygen conditions under which biodegradation would proceed at these varying depths. **Figures 5**, **6** highlight the variation in the response of the microbial community to the crude oil amendment based on oxygen concentration and temperature. These differing community structures are indicative of the different metabolic strategies needed to degrade the hydrocarbons found in crude oil. Hydrocarbon degraders are typically present at low abundances in the environment; however, their presence will increase with increasing concentrations of hydrocarbons (Head et al., 2006). This trend was also observed in our experimental microcosms. The impact of crude oil amendment increased the relative abundance of different hydrocarbon-degrading taxa in our experiment microcosms when compared to the control microcosms with no oil.

**Figures 5**, **6** show evidence of microbial bloom and succession in the experimental microcosms where the relative abundance of different taxa changed during days 5 to 15 in both oxic and hypoxic microcosms. Community dynamics shifted as the microbial community jointly acted on the different hydrocarbons found in crude oil (Hazen et al., 2016; Varjani and Upasani, 2017; Xu et al., 2018). As the preferred substrates are consumed the microbes specialized to metabolize those compounds exhaust their carbon source and are replaced by microbes able to utilize the remaining compounds resulting in a cycle of bloom and succession (Dubinsky et al., 2013; Hazen et al., 2016; Ribicic et al., 2018b). Secondary consumers are also necessary to metabolize the daughter products from the crude oil which contributes to this microbial network (Head et al., 2006).

All microcosms showed the presence of some hydrocarbon-degrading phyla, which have been previously identified in other oil-contaminated environments, most noticeably after the DeepWater Horizon oil spill (Mason et al., 2012; Dubinsky et al., 2013; King et al., 2015; Hazen, 2018). The greatest biodiversity was observed in microcosms without any crude oil amendment in both oxic and hypoxic conditions. This is unsurprising as some hydrocarbons and their intermediates are toxic to some microorganisms and the action of hydrocarbon-degraders is not induced until carbon enrichment occurs (Xu et al., 2018). The shift in community structure by day 5 shows that an influx of hydrocarbons will affect biodiversity regardless of oxygen condition. The observed reduction in biodiversity is due to the selection pressure for hydrocarbon-degraders after crude oil enrichment in these microcosms. Additionally, significant differences were noted in community structures in oxic versus hypoxic conditions. This was further indicated by our NMDS plot (**Figure 7**), where there is a clear distinction in the clustering of samples based on oxygen condition and crude oil amendment. **Figure 7** shows a difference in communities in our experimental microcosms at various time points. Initial sampling points are clustered closer together, indicating the early microbial communities share some similarities; however, after day 5, this community changes significantly. Later time points are clustered further away from each other, and distinct groupings between the oxic and hypoxic samples can be observed (**Figure 7**).

Greater biodiversity was observed in oxic microcosms. This may be explained by the typical aerobic degradation pathway of hydrocarbons which involves an oxidative reaction via oxygenase enzymes. This initial oxidation causes the hydrocarbon to become more water soluble and introduces a new reaction point for other species (Hassanshahian and Cappello, 2013). In oil-spiked oxic microcosms, there was an increased presence of hydrocarbonoclastic bacteria, which are often described as obligate hydrocarbon degraders (**Figures 5A, B**). Alkane-degrading families, such as *Alcanivorax* and *Flavobacterium*, and PAH-degrading strains, such as *Micrococcales* and *Oceanicola*, could indicate the biodegradation of the different hydrocarbon fractions found in crude oil. In addition to the increased biodiversity, aerobic biodegradation has a lower energy input for a higher energy output when compared to anaerobic biodegradation of hydrocarbons (Olajire and Essien, 2014). These factors could play a role in the hydrocarbon degradation patterns measured by the GC-MS where the relative abundance of aliphatic and aromatic hydrocarbons were significantly reduced by day 115 (**Figures 8**, **9**).

Anaerobic biodegradation of crude oil in the Caspian Sea is impacted by its environmental conditions: salinity, hypoxic to anoxic conditions and low temperatures. These harsher conditions may hinder the remediation potential of microbes by slowing their rate of metabolism. Colder temperatures also increase the viscosity of crude oil, making it more difficult to be solubilized and degraded (Das and Chandran, 2011; Ribicic et al., 2018a). Under these conditions, the anaerobic biodegradation of hydrocarbons may be incomplete, resulting in the accumulation of daughter products or intermediates (Parisi et al., 2009; Meckenstock et al., 2015). The complexity of anaerobic biodegradation is especially highlighted with PAHs. Their low solubility and stable ring structures make these compounds less bioavailable (Boll and Estelmann, 2018). Unlike aerobic biodegradation, which relies on the presence of oxygen as a terminal electron acceptor, anaerobic biodegradation uses alternative electron acceptors such as nitrate or sulfate (**Figure 3**). Taxa related to sulfate reduction (*Desulfocapsaceae*, *Desulfovibri*) and nitrogen cycling (*Nitrosococcales*) were found in hypoxic microcosms spiked with crude oil (**Figure 6B**). These bacteria are possibly coupling these metabolic processes while using carbon as an energy source, indicating that anaerobic biodegradation may be occurring. Sulfate-reducing bacteria can degrade a wide range of substrates including alkanes, benzene, and PAHs (Mahmoudi et al., 2015). Our findings are consistent with other studies at the lower depths of the Caspian who also reported the dominance of *Gammaproteobacteria* in addition to the presence of sulfate-reducing taxa and ammonia-oxidizers (Mahmoudi et al., 2015; Hazen and Techtmann, 2018). Psychrophilic and oil-degrading taxa, such as *Colwellia* and *Cycloclasticus*, were primarily found in hypoxic microcosms which were kept at low temperatures (**Figure 6B**). The presence of hydrocarbon-degrading taxa after oil amendment should signal that degradation is occurring. **Figure 8** shows evidence of the decrease in relative abundance of aliphatic hydrocarbons. The dampening of peaks within the GC-MS spectra and an increase in the unresolved complex mixture (UCM) in **Supplementary Figure 2** might also indicate biodegradation of the oil.

Although we identified the presence of PAH-degrading taxa, reduced biodiversity coupled with the complexities of anaerobic biodegradation of PAHs may contribute to the inconclusive trend noticed in **Figure 9**. The increase in the relative concentration of naphthalene, its alkylated homologs, and cycloalkane intermediates suggests the possible occurrence of biotransformation of crude oil rather than complete degradation within these microcosms (Meckenstock et al., 2004). Incomplete biodegradation of aromatic hydrocarbons could occur under these hypoxic conditions leading to the production of daughter products or dead-end metabolites which can inhibit biodegradation (Nzila, 2018). These products typically have similar structures to their parent compound causing difficulties during separation and identification using the GC-MS (Annweiler et al., 2002; Marshall and Rodgers, 2008; Nzila, 2018). These results are contrary to a previous study carried out by previous members of this group. Miller et al. (2019) found that rates of anaerobic biodegradation exceeded those of aerobic biodegradation in Caspian seawater microcosms amended with oil and a dispersant over a time period of seventeen (17) days. Dispersants can enhance the biodegradation of crude oil by reducing the surface tension to cause the formation of smaller oil droplets. These smaller droplets provide a larger surface area for microbial interaction (National Academies of Sciences and Medicine, 2020). The “bottle effect” due to long travel and holding times during the global pandemic may have also played a role in these differing results. However, this warrants further study into the mechanisms behind anaerobic degradation within this environment.

Indicator species can be used as ecological indicators of habitat types or environmental conditions due to their niche preferences. They are usually determined by analyzing the relationship between the observed species (based on abundance data) in a set of sampled sites and a user-defined classification of those sites. (De Cáceres et al., 2010). We wanted to further elucidate the microbial response to our oxygen conditions in the presence of crude oil. The identification of halotolerant species in both oxic and hypoxic indicates the adaptation of the indigenous microbes to the brackish conditions within the Caspian. Oxic microcosms (**Table 1**) show the presence of aerobic bacteria previously correlated to hydrocarbon degradation. Hypoxic microcosms (**Table 2**) were more correlated with the presence of mixotrophs and psychrophilic taxa which could indicate possible microaerophilic conditions in these microcosms. Taxa involved in sulfate cycling were also more correlated with hypoxic microcosms, potentially highlighting a metabolic strategy for hydrocarbon degradation via an alternate terminal electron (**Figure 3**). Notably, our indicator species analysis also identified an ASV associated with the unclassified *Gammaproteobacteria* (stat = 0.885) that dominated hypoxic microcosms.

## 5 Conclusion

Microcosms showed greater similarity to each other based on oxygen concentration as opposed to hydrocarbon amendment. The variation in metabolic strategies needed to degrade oil in oxic and hypoxic conditions could account for these differences. The microbes within assembled microcosms showed some adaptation to their simulated environmental conditions and displayed degradation of hydrocarbon fractions found within crude oil. Distinct communities were present in surface waters and deeper waters of the Caspian Sea. These communities have been influenced by the continuous presence of oil in the water body and contain a mix of different microorganisms that have been shown to degrade hydrocarbons themselves or have been found in other hydrocarbon-enriched environments. The structure of these communities changed over time after the crude oil amendment, which was indicative of a pattern of bloom and succession typically noticed in places that have experienced an oil spill. Metagenomic analysis of these microcosms is pending to further elucidate the possible functional role of the identified species to crude oil amendment in differing oxygen conditions.

## Data availability statement

The original contributions presented in the study are included in the article/**Supplementary Materials**, further inquiries can be directed to the corresponding author/s.

## Author contributions

ZG: Formal analysis, Investigation, Methodology, Visualization, Writing – original draft, Writing – review & editing. AP: Data curation, Software, Writing – review & editing. JM: Methodology, Resources, Conceptualization, Software, Writing – review & editing. MFC: Formal analysis, Investigation, Resources, Conceptualization, Writing – review & editing. DJ: Funding acquisition, Project administration, Resources, Writing – review & editing. OP: Funding acquisition, Project administration, Resources, Writing – review & editing. NG: Investigation, Project administration, Resources, Supervision, Writing – review & editing. MC: Formal analysis, Investigation, Methodology, Writing – review & editing. PG: Formal analysis, Investigation, Resources, Writing – review & editing. TCH: Conceptualization, Funding acquisition, Investigation, Methodology, Project administration, Resources, Supervision, Visualization, Writing – original draft, Writing – review & editing.
