# Supplementary figures and images for: Comparing the response of the indigenous microbial community to crude oil amendment in oxic versus hypoxic conditions
Source: Front Microbiomes. 2023 Dec 14;2:1270352. doi: 10.3389/frmbi.2023.1270352 (PMC12993514; doi:10.3389/frmbi.2023.1270352)

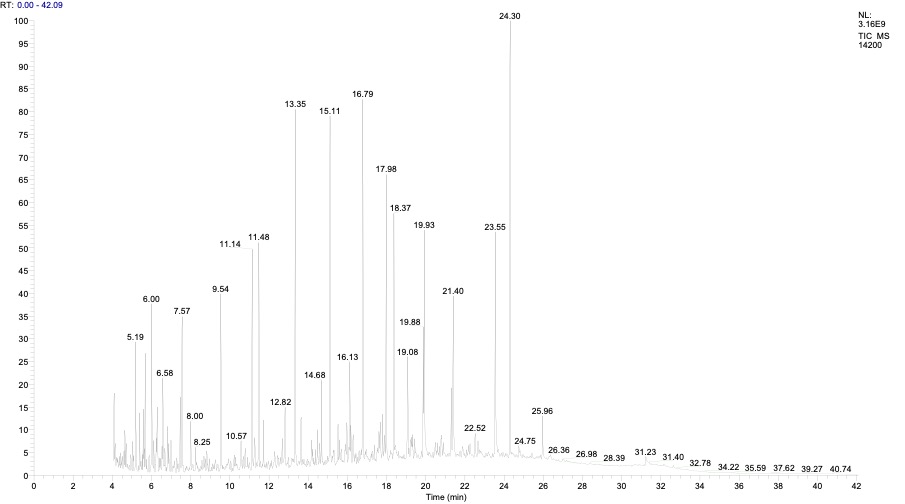

Supplement: Supplementary file 1 [file Image_1.jpeg]

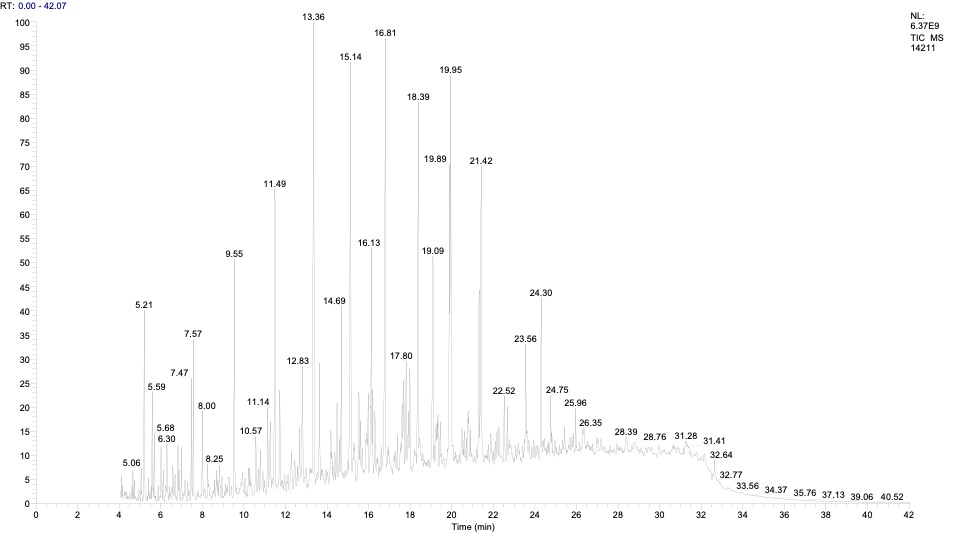

Supplement: Supplementary file 2 [file Image_2.jpeg]

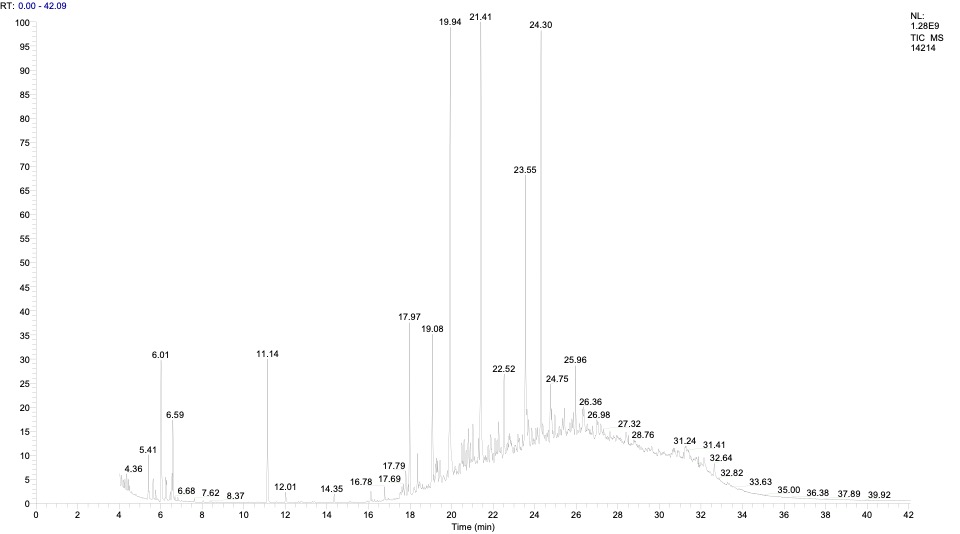

Supplement: Supplementary file 3 [file Image_3.jpeg]

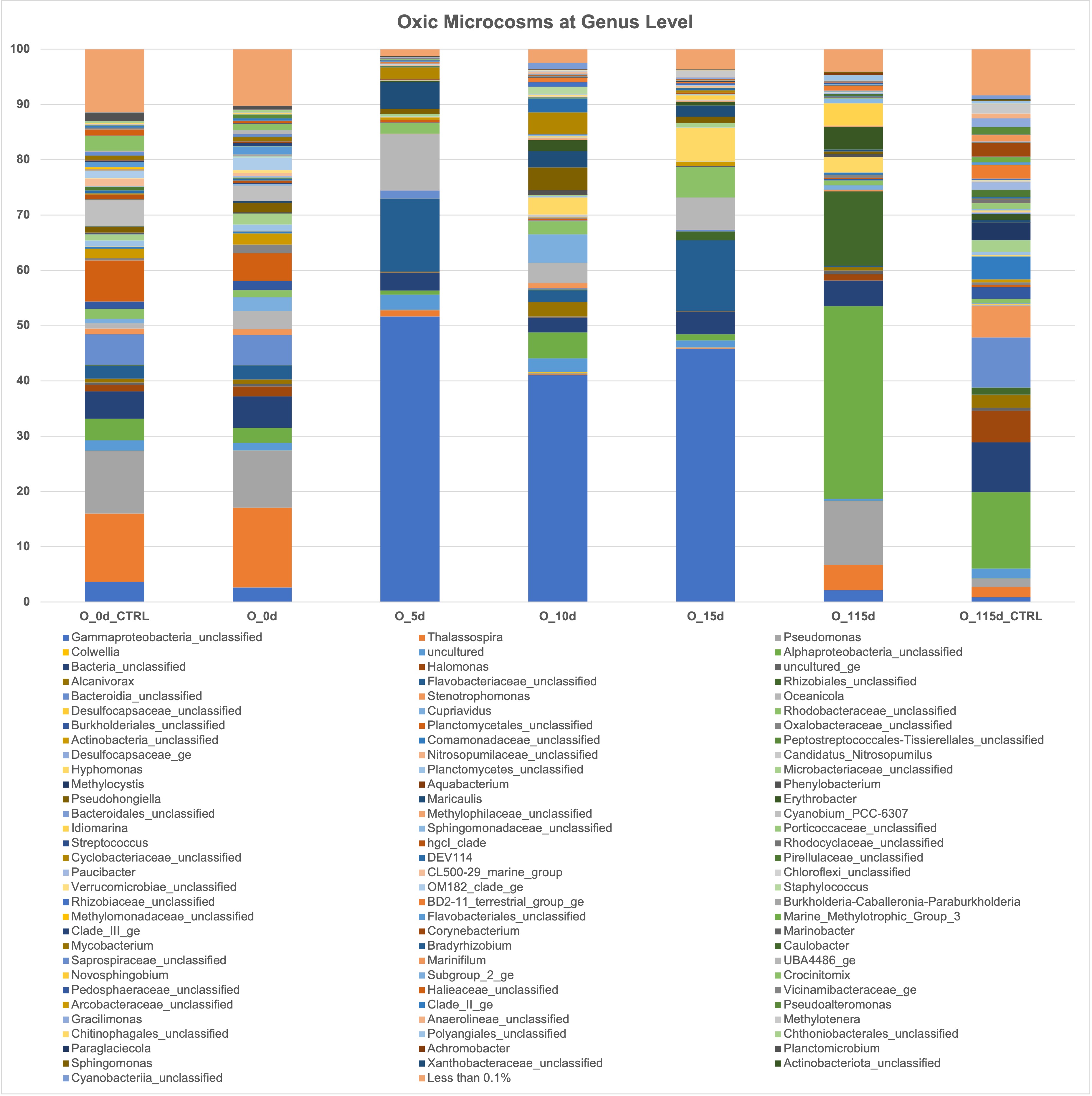

Supplement: Supplementary file 4 [file Image_4.jpeg]

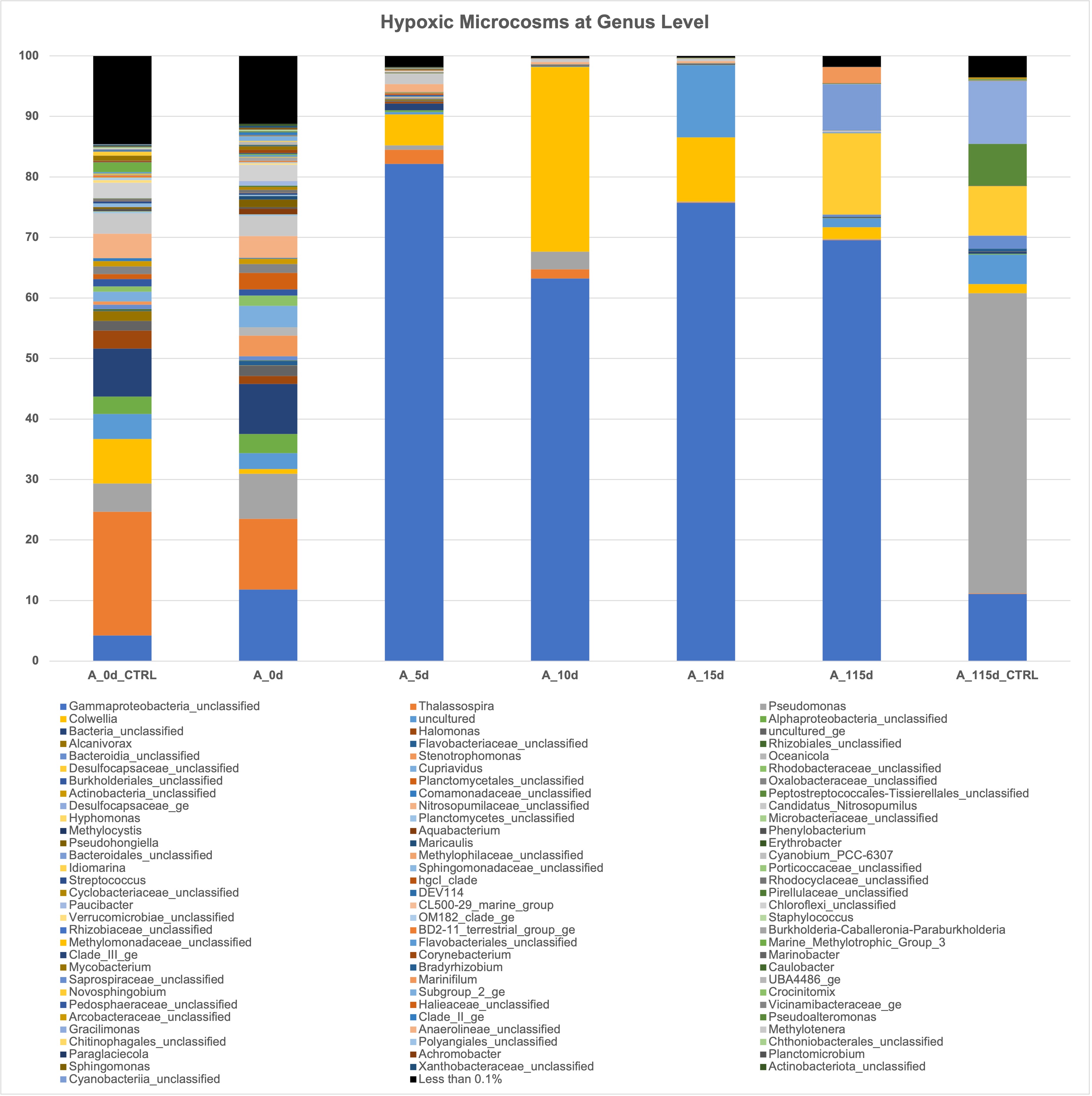

Supplement: Supplementary file 5 [file Image_5.jpeg]
