# Supplementary material for: Comparing the response of the indigenous microbial community to crude oil amendment in oxic versus hypoxic conditions
Source: Front Microbiomes. 2023 Dec 14;2:1270352. doi: 10.3389/frmbi.2023.1270352 (PMC12993514; doi:10.3389/frmbi.2023.1270352)
Supplement: Supplementary file 6 [file Table_1.docx]

**Supplemental Data**

Supplemental Figure 1: Full scan GC/MS chromatogram for the Caspian Oil Standards used in the microcosms. m/z 50-450.

Supplemental Figure 2: Full scan GC/MS acquisition of the sample extract from the hypoxic microcosm at test end (115 days) - m/z 50 to 450.

Supplemental Figure 3: Full scan GC/MS acquisition of the sample extract from the oxic microcosm at test end (21 days) - m/z 50 to 450.

Supplemental Figure 4: Showing relative abundance of bacterial genera in oxic microcosms.

Supplemental Figure 5: Showing relative abundance of bacterial genera in hypoxic microcosms.

Supplemental Table 1: Initial environmental parameters of Caspian seawater based on depth. Water was collected from 25m and 350m for experiment microcosms.
